# Dynamic Tensile Response of Basalt Fibre Grids for Textile-Reinforced Mortar (TRM) Strengthening Systems

**DOI:** 10.3390/polym17020132

**Published:** 2025-01-08

**Authors:** Amrita Milling, Giuseppina Amato, Su Taylor, Pedro Moreira, Daniel Braga

**Affiliations:** 1 School of Natural and Built Environment, Queen’s University Belfast, David Keir Building, Stranmillis Road, Belfast BT9 5AG, UK; 2 Institute of Science and Innovation in Mechanical and Industrial Engineering (INEGI), FEUP Campus, Rua Dr. Roberto Frias 400, 4200-465 Porto, Portugal

**Keywords:** dynamic, strain rate, basalt fibre, tensile tests, textile reinforcement, scanning electron microscopy, computer vision, TRM, FRCM

## Abstract

The present work constitutes the initial experimental effort to characterise the dynamic tensile performance of basalt fibre grids employed in TRM systems. The tensile behaviour of a bi-directional basalt fibre grid was explored using a high-speed servo-hydraulic testing machine with specialised grips. Deformation and failure modes were captured using a high-speed camera. Tensile strain values were extracted from the recorded images using the MATLAB computer vision tool, ‘vision.PointTracker’. The specimens, consisting of one and four rovings, were tested at intermediate (1–8/s) and quasi-static (10^−3^/s) strain rates. After the tensile tests, scanning electron microscopy (SEM) analyses were performed to examine the microscopic failure of the material. Linear and non-linear stress–strain behaviours were observed in the range of 10^−3^ to 1/s and 4 to 8/s, respectively. Tensile strength, ultimate strain, toughness, and elastic modulus increased at intermediate strain rates. Overall, the dynamic increase factors for these properties, except for the latter, were between 1.4 and 2.3. At the macroscopic level, the grid failed in a brittle manner. However, microscopic analyses revealed that the failure modes of the fibre and polymer coating were strain-rate sensitive. The enhanced tensile performance of the grid under dynamic loading conditions rendered it suitable for retrofitting structures prone to extreme loading conditions.

## 1. Introduction

Textiles are the primary reinforcement in two advanced techniques for strengthening civil engineering structures: externally bonded fibre-reinforced polymers (FRPs) and textile-reinforced mortars (TRMs). These state-of-the-art methods have gained significant attention for their effectiveness in enhancing structural performance and durability. In the 1990s, the FRP method emerged, utilising a composite made of a continuous, uni-directional fibre textile saturated with resin [1,2]. However, constraints related to the use of organic resins for impregnating textiles led to the introduction of the TRM method in the early 2000s. The TRM approach integrates a bi-directional, open-mesh grid into an inorganic matrix [3]. It is important to note that TRM grids are typically treated with a polymer coating to protect the fibres or enhance the bond between the grid and the cementitious matrix. Thus, similar to FRP composites, their use in elevated temperature environments is also limited [4]. Textile strengthening techniques are used in various applications, including those that expose them to extreme, dynamic conditions. They are used to reinforce structures against blasts and shockwaves [5], offshore structures exposed to harsh wave actions [6], collision-prone ship platforms and piers [7], and building/bridge components in seismic regions [8]. Textiles have also been explored as possible connections to minimise roof uplift during hurricanes [9].

Previous studies have shown that strain rate is a critical factor influencing the tensile behaviour of the textiles used for structural strengthening [10,11,12]. By understanding the rate-dependent behaviour of these materials, engineers can develop safe, reliable, and efficient structures, particularly in the dynamic loading events mentioned above. However, published work on textile reinforcements at intermediate (10^−1^ to 10^3^/s) and high (10^3^ to 10^6^/s) strain rates is lacking and inconclusive.

Most dynamic characterisation efforts were carried out using a drop-weight impact system that applied intermediate strain rates of 40 to 200/s. Ou et al. [10] investigated basalt yarn and basalt FRP (BFRP) composites in the range of 40 to 160/s. Basalt yarns demonstrated increases in elastic modulus (62.9%) and tensile strength (32.2%) with an increasing strain rate. However, its maximum strain and toughness initially increased in the range of 40 to 80/s before decreasing by 12.5 and 9.8%, respectively, at 160/s. The BFRP composites displayed a modest increase in the elastic modulus (8.2%) and tensile strength (3.8% up to 120/s), followed by a slight strength reduction of 2.2% at 160/s. By contrast, maximum strain increased by 13.8% across the full strain rate range. Despite exhibiting a lower elastic modulus, the BFRP composites outperformed the basalt yarn in terms of tensile strength, maximum strain, and toughness at equivalent strain rates. The basalt yarns failed due to fibre breakage, whereas the BFRP composites exhibited distinct failure patterns depending on the strain rate. At 40/s, the damage remained localised with minimal fibre pullout. As the strain rate increased to 160/s, the failure zone expanded across the gauge-length region with extensive fibre pullout. Bai et al. [11] examined polyethylene terephthalate (PET) fibre bundles in the range of 1/600 to 160/s. Bilinear stress–strain curves were reported for all samples. The initial elastic modulus (E1) increased from 11 GPa at 1/600 to 17.3 GPa at 160/s, while the second elastic modulus (E2) showed a similar trend, increasing from 5.8 to 10.8 GPa. Over the same range, tensile strength increased by approximately 33%, while ultimate strain decreased from 13 to 9.1%. The failure modes transitioned from dispersed, successive fibre ruptures and uneven fracture surfaces at 1/600/s to a more uniform and simultaneous fibre failure with smoother fracture surfaces under dynamic loading. Ou et al. [13] also studied the tensile properties of Kevlar^®^ 29 single filaments and yarns in the range of 1/600 to 160/s. An increase in the strain rate resulted in a 28.9 and 32.8% increase in the elastic modulus and tensile strength, respectively. However, the ultimate strain decreased from 2.6 to 2.2%. The tensile strengths of the single filaments were significantly higher than those of the yarns, indicating a pronounced structural-scale effect. Ou et al. [14] investigated the effect of the strain rate on the mechanical properties of uni-directional glass FRP (GFRP) composites and individual yarns in the range of 1/600 to 200/s. As the strain rates increased from quasi-static to dynamic, the GFRP composite and yarn exhibited an increase of 49.1 and 88% in tensile strength, respectively. The ultimate strain and toughness initially improved with increasing strain rates but decreased at 120 to 160/s.

Yao et al. [15] utilised an MTS high-rate servo-hydraulic testing machine to investigate the behaviour of several types of plain-woven, uni-directional textiles, including aramid, glass, basalt, and carbon in the range of 25 to 100/s. The tensile strength increased across all materials with the increasing strain rate, with basalt exhibiting the highest relative increase of 59%, from 1095 to 1743 MPa. Carbon, glass, and aramid textiles demonstrated increases of 30, 36, and 25%. Trends in elastic modulus were inconsistent, with basalt initially increasing but declining at higher rates and carbon showing a steady decrease from 147 to 88.6 GPa. The ultimate and maximum strains exhibited overall increases with the strain rate; basalt textiles increased from 2.36 to 3.24%, while carbon, glass, and aramid displayed varying degrees of enhancement. The failure modes of these materials were reportedly independent of the strain rate, and glass textiles were found to be the most ductile.

In addition, Zhu et al. [16] investigated basalt filament tows at intermediate and high strain rates using a split Hopkinson tension bar. Tows were tested between 600 and 3000/s and exhibited variations in mechanical properties with increased strain rates. A stiffer response was observed under dynamic loading, as the elastic modulus increased from approximately 38 to 48 GPa. Similarly, the tensile strength increased from 1200 to 1900 MPa. However, the ultimate strain decreased from 3.8 to 2.8%. Fractographic analyses revealed a combination of ductile and brittle characteristics at 600/s. In contrast, at 3000/s, the failure mode became predominantly brittle, with severe damage near the incident bar.

All of the studies described above relate to uni-directional textiles associated with the FRP method. The dynamic behaviour of bi-directional, open-mesh grids used in textile-reinforced concrete or mortar remains largely unexplored. Zhu et al. [12] considered the tensile behaviour of alkali-resistant (AR) glass, polyethylene (PE), and carbon TRM grids within the range of 9–28/s. The glass and carbon grids demonstrated linear elastic behaviour with brittle failure, whereas the PE material exhibited ductile behaviour. A recent study by Milling et al. [17] on basalt TRM grids was conducted at 0.2 and 2/s. This study reported how the elastic modulus and tensile strength of the material decreased with an increase in the strain rate. Both studies reported that specimens deemed suitable for quasi-static testing were unsuitable for dynamic loading conditions due to premature failures.

The strain-rate sensitivity of textiles is highly dependent on the fibre type, roving orientation, mesh size, resin properties, and the performance of fibre–resin interfaces [18,19,20]. Nevertheless, the relationship between these parameters and the tensile mechanical characteristics under dynamic strain rates remains ambiguous. Also, knowledge of the experimental approach for investigating this material under dynamic loading conditions is lacking. Specimen types and gripping procedures that have been successful in FRP uni-directional textiles and TRM tests under quasi-static conditions cannot be transferred to TRM grids under dynamic loading [12,17]. Moreover, the effect of the specimen size on the dynamic tensile properties of TRM grids is yet to be explored. These gaps highlight the need for research efforts to advance knowledge and develop standardised dynamic testing guidelines, which are currently non-existent.

This study investigated the tensile characteristics of a basalt TRM grid at quasi-static (10^−3^/s) and intermediate (1–8/s) strain rates. A basalt fibre grid was selected because of the increasing preference for basalt fibres over carbon and glass fibres in TRM systems [21]. Basalt fibres possess high tensile strength, fatigue resistance, and thermal resistance up to 650 °C [22,23]. They are manufactured without harmful additives, making their production environmentally friendly [23]. Compared to carbon fibres, basalt fibres are more cost-effective and require significantly less energy, thus reducing their environmental impact [24]. Moreover, they surpass glass fibres in terms of recyclability, thermal resistance, and corrosion resistance [25,26]. The choice of the grid geometry was influenced by product availability and studies that validated the mechanical performance of TRM composites made with grids of similar 6 mm × 6 mm mesh sizes [27,28,29].

Tensile tests were performed using a high-speed servo-hydraulic machine with an improved version of the specialised grips used in [17]. The deformation and failure modes during tensile loading were captured with a high-speed camera. Strain analysis was performed by applying the ‘vision.PointTracker’ from MATLAB’s Computer Vision Toolbox, which extracted displacement values by pixel tracking. The experimental methodology detailed the types of grips and specimens explored and the challenges encountered. This study provides novel insights into how variations in the strain rate and specimen geometry influence the stress–strain behaviour, tensile mechanical properties, and failure modes of the basalt TRM grid. The dynamic increase factors for the tensile strength, ultimate strain, and toughness were also determined, and empirical equations for predicting these properties at various strain rates were proposed. Furthermore, for the first time, scanning electron microscope (SEM) analysis was performed to provide insights into the microscopic failure of the basalt TRM grid under dynamic conditions.

## 2. Materials and Methods

### 2.1. Materials

**Figure 1:**
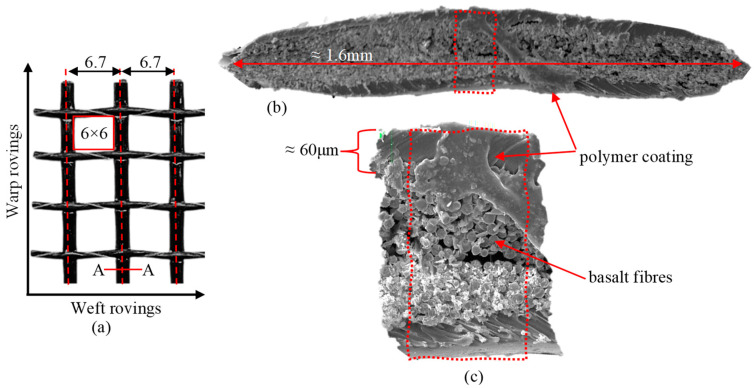
(**a**) Photo of the basalt grid with three rovings in the warp direction (dimension in mm); (**b**) ×100 magnified cross-section A-A of a warp roving; and (**c**) ×500 section view of warp roving.

The MAPEGRID B 250 basalt fibre grid utilised in this study was manufactured by MAPEI (Milan, Italy). The grid shown in Figure 1a comprises orthogonal warp and weft rovings, forming a 6 mm × 6 mm squared mesh pattern. The centre-to-centre spacing between the rovings is approximately 6.7 mm. Figure 1b,c illustrate the cross-section and distribution of fibres and polymer coating in a representative roving. The fibres are coated by the manufacturer with an anhydrous, alkali-resistant polymer to protect them from chemical attacks when embedded in a cementitious matrix. The material has an equivalent thickness of 0.039 mm, a mass density of 2.75 g/cm^3^, and a weight of 250 g/m^2^. The tensile strength is 60 kN/m, with an elongation of 1.8% and an elastic modulus of 89 GPa.

### 2.2. Tensile Testing

All tensile tests were conducted at the Institute of Science and Innovation in Mechanical and Industrial Engineering (INEGI), Portugal, using a high-rate servo-hydraulic testing machine that could operate within the range of 1 to 1100 mm/s with a load capacity of 20 kN. Deformation and failure behaviours were captured with a high-speed camera (Photron FASTCAM Nova S6 type, model 800K-M-16 GB 10GbE made in Yonezawa, Japan) and two halogen spotlights. Twelve ‘grid’ (four warp rovings) specimens and six ‘roving’ (one warp roving) specimens were tested at strain rates in the range of 10^−3^ and 8/s following a set of trial setups.

#### 2.2.1. Tensile Grips and Specimen Types

**Figure 2:**
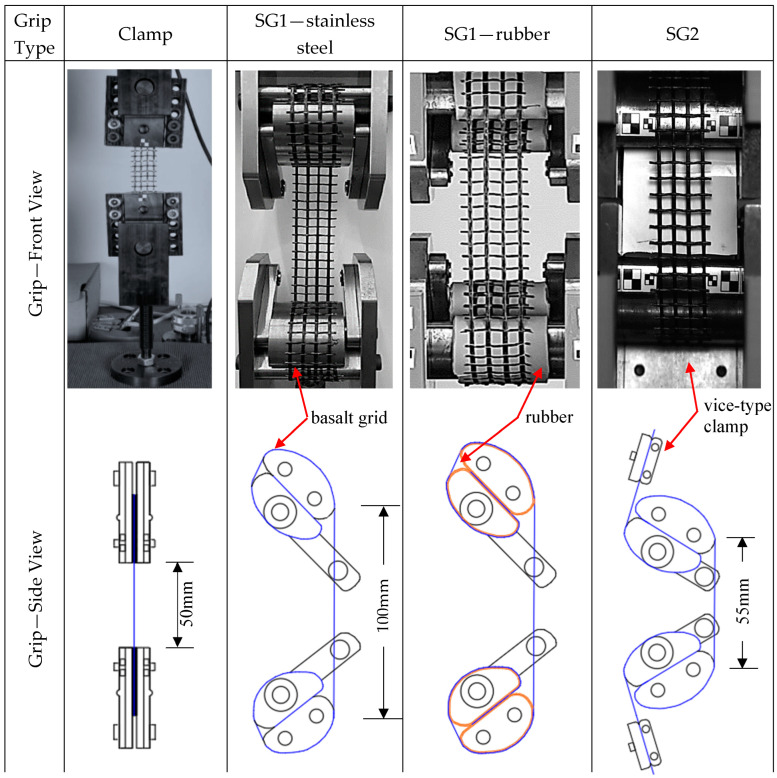

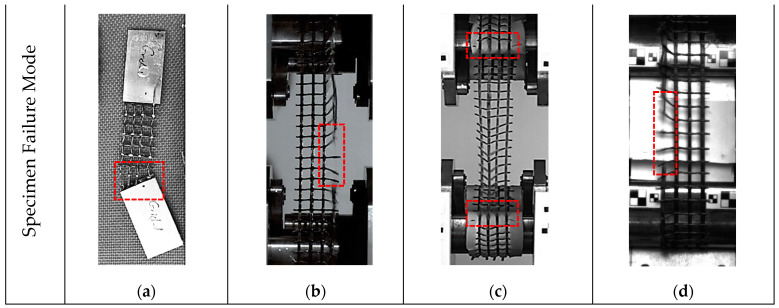
Front view, side view, and failure modes of basalt grid specimens tested with (**a**) clamp grips; (**b**) specialised grips 1 (SG1); (**c**) SG1 with rubber; and (**d**) specialised grips 2 (SG2). The red boxes highlight the regions of failure.

Due to the lack of standardised dynamic testing methods for grids used in TRM applications, this study explored several types of grips and specimens to achieve reliable results (see Figure 2). Initially, trial tests were conducted with clamp grips and aluminium-tabbed coupon specimens of the length 140 mm and four warp rovings. This specimen type was adopted from [29], which established that this specimen effectively distributed the gripping load and prevented specimen failure at the gripping areas under quasi-static loading. However, after numerous attempts, the specimens failed due to the sliding of one or more rovings from the gripping section, as shown in Figure 2a.

A set of specialised grips made of stainless steel (SG1) was developed to ensure valid specimen failure. SG1 specimens were cut to 490 mm lengths and spanned a width of four warp rovings. They were aligned between the top and bottom grips with a gauge length of 100 mm. The specimen folded over the grips and ended between the two jaws, as shown in Figure 2b. With increasing tension placed on the specimen, the clamping force increased. No signs of stress concentrations were observed in the grip area; nonetheless, using SG1 led to excessive grip slippage and failure to reach the desired strain rates, as discussed in [17]. To address these problems, a 2 mm thick rubber layer was added to the grip region of SG1 to enhance contact friction, as shown in Figure 2c. Wrapping the contact area of SG1 with rubber proved ineffective, leading to non-uniform stress distribution and premature failure at the grips. Discontinuities in the stress–strain behaviours were also evident when the grip area was partially or fully covered with rubber, indicating that the specimens were not appropriately fastened.

**Figure 3:**
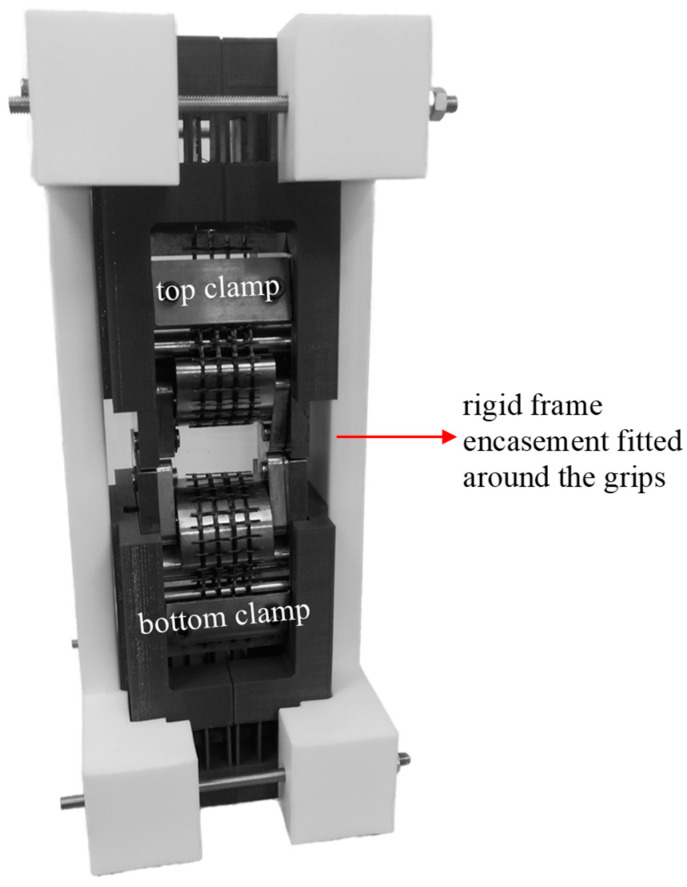
Image of the specialised grips SG2 encased in a rigid frame to help maintain the planar configuration of the samples.

The specialised grips, SG2, were developed through modifications made to SG1. Vice-type clamps were installed at the top and bottom of the grips to prevent the ends of the specimens from slipping, as shown in Figure 2d. The distance between the top and bottom grips was also reduced from 100 to 55 mm. These alterations resolved the slippage issue and made it possible to achieve the targeted strain rates. SG2 specimens were cut at lengths of 490 mm and had a width of one or four warp roving(s), depending on whether they included four or one warp roving, respectively. The specimen installation process involved the following steps: (i) securing the upper end of the specimen using the top clamp; (ii) looping the loose end around the curved grips and threading it through the jaws; and (iii) securing the lower end of the specimen using the bottom clamp (see Figure 3). Once a sample was secured, it was subjected to the weight of the lower grip and the associated impact disc, which applied a pre-tension load of approximately 63 N. The pre-tension helped remove any visible slack in the samples, ensuring they were in a planar state before testing began. The tensile grips were also encased in a rigid frame, as shown in Figure 3, to prevent any movement or distortion of the grips and the sample during high-speed loading.

#### 2.2.2. Testing Programme

The tensile load was recorded at 750 kHz, and images were captured at 6.4 kHz for all tests, except for those conducted at 1 mm/s, which were recorded at 50 Hz. Three samples were tested at each loading rate, as listed in Table 1, with the exception of 800 mm/s. Specimens were labelled ‘G’ and ‘R’ for four-roving and single-roving specimen sizes, respectively. The second component of the specimen ID is the measured strain rate (10^−3^, 1, 4, 5, and 8/s) derived from the slope of the strain–time graphs, and the last component represents the sample number. Notably, the grid specimens reached lower strain rate values than the single rovings at the same loading rate of 1100 mm/s (see G5 and R8 specimens).

#### 2.2.3. Strain Data Processing

**Figure 4:**
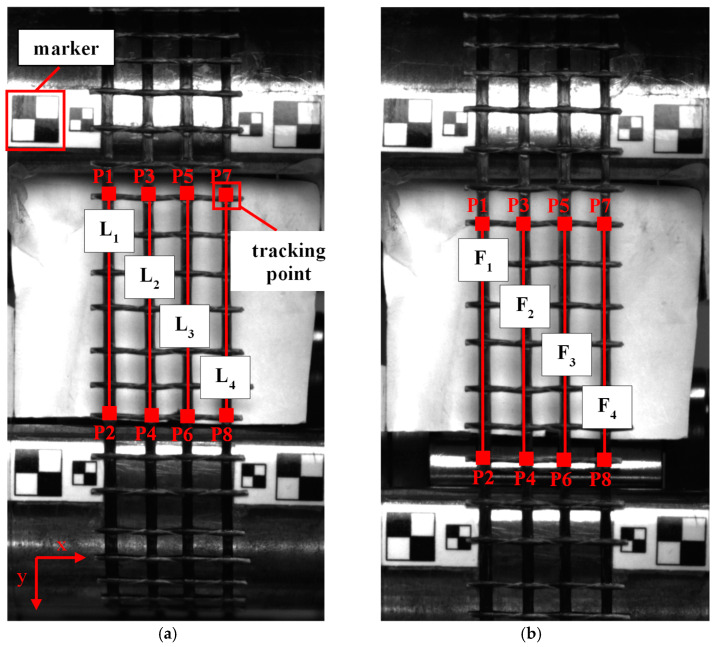
An illustration of the four virtual extensometers on the grid specimen used to determine strain: (**a**) initial frame; (**b**) frame at maximum tensile load.

A modified version of the MATLAB pixel tracking code developed by Lydon et al. [30] was used to quantify the strain. The computational analysis was performed with MATLAB version 9.13.0.2126072 (R2022b) Update 3.The sequence of images for each specimen was first converted to grayscale to reveal the intensity information and easily track the points of interest. This method was convenient as it did not require a speckle pattern or ‘surface component’ as required by commercial digital image correlation software. For the grid and roving specimens, virtual extensometers were defined in the first frame by selecting eight points (P1–P8) or two points (P1–P2) of interest, respectively. Figure 4 illustrates the placement of these points at the centre of a grid specimen. The vertical displacements of the points, measuring 15 × 15 pixels, were tracked from the first frame (Figure 4a) and continued across the sequence to the frame corresponding to the maximum tensile load (Figure 4b) by implementing the vision.PointTracker function.

The strain values were derived from the displacement measured in the gauge length region of the specimens to avoid the effects of friction and slippage in the grip region [31]. The strain (*ε*) was computed as the average of the individual strains measured by each virtual extensometer, as expressed in Equation (1):

(1) $$\varepsilon =\frac{1}{N}\sum_{i=1}^{N}\left(\frac{F_{i}-L_{i}}{L_{i}}\right)$$

where *N* represent the number of extensometers and *F_i_* and *L_i_* represent the original and final lengths of the *i*-th extensometer, respectively. Pixel-based measurements of the original and final lengths were converted into millimetres by determining the image resolution (pixels per mm) using MATLAB’s Imtool. Calibration was facilitated by placing square markers of predetermined dimensions on the grips.

#### 2.2.4. Stress Evaluation

The tensile stress was determined by dividing the applied force by the cross-sectional area of the specimen. Given the discontinuous distribution of fibres across the width of the samples, the cross-sectional area was calculated as a product of the grid’s equivalent thickness (0.039 mm) and width [29]. The width was obtained by multiplying the number of warp rovings (4 for grid specimens and 1 for single-roving specimens) by the spacing between the warp rovings.

### 2.3. Microscopic Analysis

After tensile testing, the microstructures of the samples obtained from the ends of the ruptured rovings were analysed. The samples were no longer than 12.5 mm, which was the diameter of the SEM specimen stubs. The specimens underwent gold plating using a manual Agar sputter coater and were then examined using a Quanta FEG-250 scanning electron microscope (Beverly, MA, USA). Depending on the sample, the microscope was operated at either 10 kV or 20 kV accelerating voltage to mitigate the impact of charging.

## 3. Results and Discussion

### 3.1. Strain Rate Effect on the Stress–Strain Relationship

**Figure 5:**
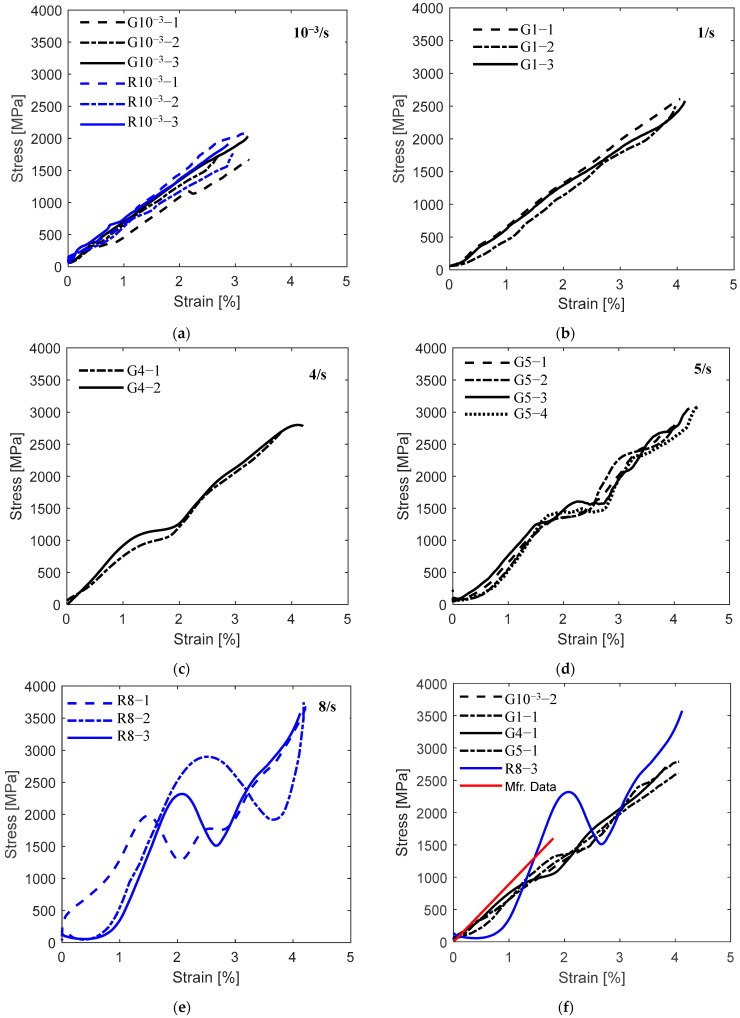
Stress–strain relationship of the basalt fibre grid at various strain rates: (**a**) 10^−3^/s; (**b**) 1/s; (**c**) 4/s; (**d**) 5/s; (**e**) 8/s; and (**f**) representative samples from each strain rate.

The stress–strain curves of the grid and roving samples at various strain rates are presented in Figure 5. The comparison between the five groups of samples shows how the curve trends change from linear to non-linear with increasing strain rates. The stress–strain relationship of all 10^−3^ and 1/s specimens, except G10^−3^-1, displayed one linear elastic region until failure. Specimens G4 and R8-2, as shown in Figure 5b,e, exhibited two linear regions, while specimens G5, R8-1, and R8-3 had three linear regions, as shown in Figure 5c,d. Figure 5f shows the stress–strain curves of a representative sample from each strain rate alongside the manufacturer’s data (Mfr.Data) curve. The basalt grid demonstrated superior performance at strain rates of 10^−3^–5/s compared to the manufacturer’s specification. The G samples demonstrated relatively consistent trends, with elastic regions initially aligning with the manufacturer data but diverging beyond a strain of approximately 1%. The R specimen, on the other hand, exhibited distinct stress–strain behaviour with pronounced non-linearity at 8/s.

**Figure 6:**
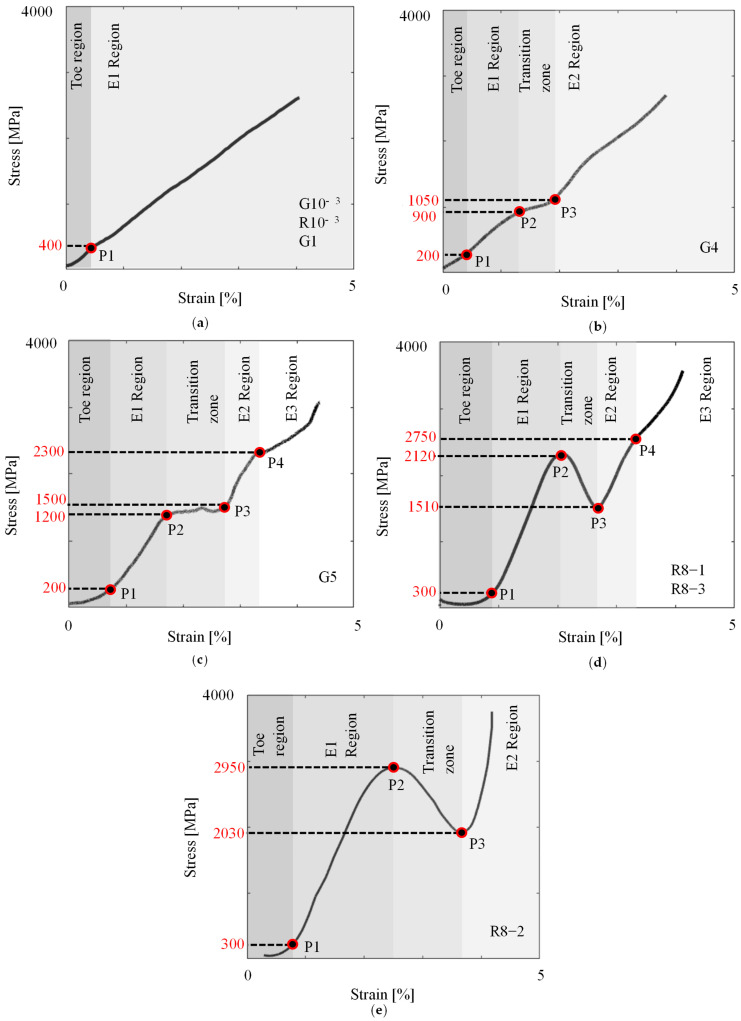
Schematic stress–strain curves highlighting the points and regions of interest for the different strain rate ranges of (**a**) 10^−3^ to 1/s; (**b**) 4/s; (**c**) 5/s; and (**d**,**e**) 8/s.

The toe, linear regions, and transition zones of the stress–strain curves are highlighted in the schematic in Figure 6. A non-linear, gradually increasing slope at the initial stage, called the ‘toe region’, was typical in all plots. The transition point (P1) between the toe and E1 region primarily occurred at approximately 400 MPa or less, except for specimen R8-1, which occurred at 1100 MPa. The specimens tested at 10^−3^ and 1/s ruptured at the end of the E1 region. However, in the range of 4 to 8/s, the specimens possessed a ‘knee’ feature at the end of the E1 region, followed by a distinct ‘transition zone’. The maximum point of the knee at P2 represents the yield point where the material started to experience irreversible fibre or polymer damage [32,33]. The yield stress increased from 900 to 2950 MPa as the strain rate increased from 4 to 8/s. The transition zones for the G and R specimens occurred within the range of 900–1500 MPa, 1.3–2.7%, and 1510–2950 MPa and 1.4–3.6%, respectively. For the G specimens, the stresses at the endpoints of the transition zones, P3, were higher than the yield stress, demonstrating a strain-hardening behaviour. Conversely, softening behaviour was noted for the R specimens, where the stress values at P3 were lower than the yield stresses. Region E2 ranged from 1050 to 3750 MPa. A modest knee between 2300 and 2750 MPa facilitated the transition to the E3 region for all specimens in the range of 5 to 8/s, except for specimen R8-2.

The observed non-linear behaviour of the basalt grid as it transitioned from regions E1 to E3 are indications of microscopic damage accumulation [34,35,36] and thermal softening [37], both of which were confirmed by the SEM analyses discussed in the ‘Microscopic Failure’ section of this paper. Furthermore, the more noticeable non-linear behaviour of the R specimens relative to the G specimens, especially in the transition zones, could be due to the load distribution mechanism. For the G specimens with several weft and warp rovings, the failure of certain fibres within the rovings resulted in the redistribution of the load to other fibres and rovings. In the R specimens, the load is borne entirely by the fibres within a single roving, which can lead to catastrophic failure if one or more fibres fail.

**Figure 7:**
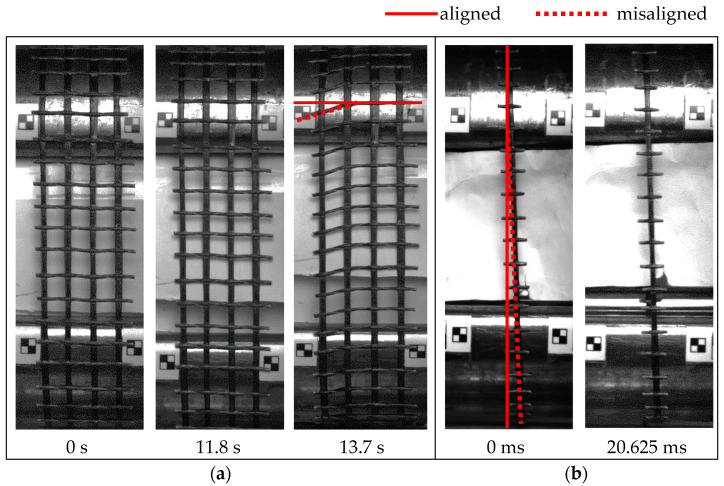
Specimen misalignment during testing: (**a**) G10^−3^-1; (**b**) R8-2.

The inconsistent stress–strain relationships displayed by specimens G10^−3^-1 and R8-2 could be linked to their misalignment during testing. Figure 7 shows that all the weft rovings for G10^−3^-1 were horizontal when the test started and began misaligning at around 13.7 s, whereas R8-2 maintained a slanted orientation for the entire test duration. Specimen G10^−3^-1’s change in alignment corresponded to a considerable decrease in the stress–strain behaviour observed in Figure 5a at a strain value of around 2.1%. This sample also exhibited the lowest elastic modulus and tensile strength of 50 GPa and 1664 MPa, respectively. Interestingly, in the case of R8-2, despite being misaligned, the tensile strength and ultimate strain of this sample were consistent with the results reported for other (aligned) roving specimens.

### 3.2. Strain Rate Effect on Tensile Mechanical Characteristics

#### 3.2.1. Tensile Strength

**Figure 8:**
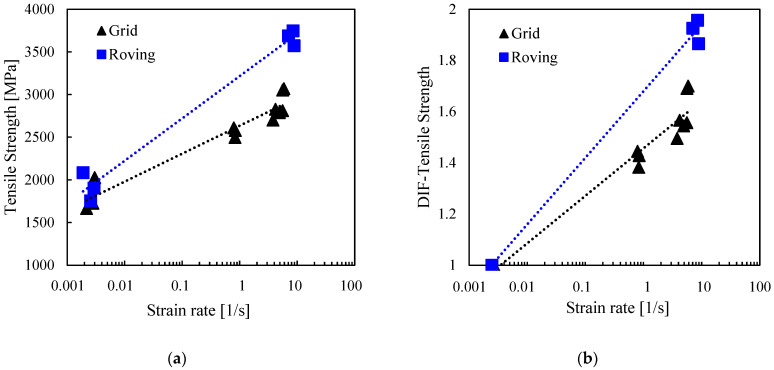
Effect of strain rate on (**a**) tensile strength; (**b**) dynamic increase factor for tensile strength.

Figure 8 shows the increase in the tensile strength and dynamic increase factor with the increasing strain rate. The tensile strength of the grid specimens increased from 1664 to 3069 Mpa (84%) when the strain rate increased from 10^−3^ to 5/s. Roving specimens also followed a similar trend, where the strength increased by 114% from 1755 (at 10^−3^/s) to 3747 Mpa (at 8/s). The results suggest that the R specimens are more than 20% stronger than the G specimens in the dynamic range. Usually, smaller specimens have a higher strength than their larger counterparts of the same material due to fewer defects and a lower probability of critical flaws [15]. However, in this study, the lower strength of the grid specimens could be attributed to the interaction between the weft rovings and non-uniform stress distribution due to their deviation from the parallelism of warp rovings. A visual inspection of the G specimens revealed substantial misalignment of up to 15^0^ and a mesh size variation from 1 to 3 mm. Single-roving specimens do not fully represent the realistic, complex behaviour of bi-directional grids and, therefore, should not be used to characterise the grid for design purposes, as this approach may lead to the overestimation of tensile strength.

The enhanced tensile strength observed at dynamic rates for both the R and G specimens may be attributed to the short duration of the deformation process, which was less than 10 ms. Under such conditions, microstructural defects, which significantly influence the tensile strength of basalt fibres [38], have little time to initiate, propagate and connect, thus requiring a higher stress to fracture [10]. Also, there may have been inadequate time for the fibres to shift and reorganise, resulting in intensified inter-fibre friction. Although there has been no confirmation regarding basalt fibres, several studies [39,40,41] have demonstrated that enhanced inter-fibre friction in polyester, Kevlar, and aramid fibres improves mechanical characteristics. The polymer coating may have also been a significant contributor to the enhanced tensile strength of the grid, as the findings of [18,42,43] revealed a positive correlation between the tensile strength and strain rate for polymer coatings. Nonetheless, the specific influence of the fibres, polymer coating, or inter-surface interactions in improving the tensile strength of the basalt grid is unknown and remains an open issue.

Similar trends of increased tensile strength with increasing strain rates were reported for basalt yarn [10], unidirectional fibres [15], and filament tows [16] at 40–160/s, 25–100/s, and 600–3000/s, respectively. The tensile strength of the basalt grid was higher than that of basalt yarn at 40–160/s [10] by more than 50%, for basalt unidirectional fabrics at 25 to 100/s [15] by more than 100%, and for basalt filament tows at 600 to 3000/s [16] by more than 80%.

#### 3.2.2. Ultimate Strain

**Figure 9:**
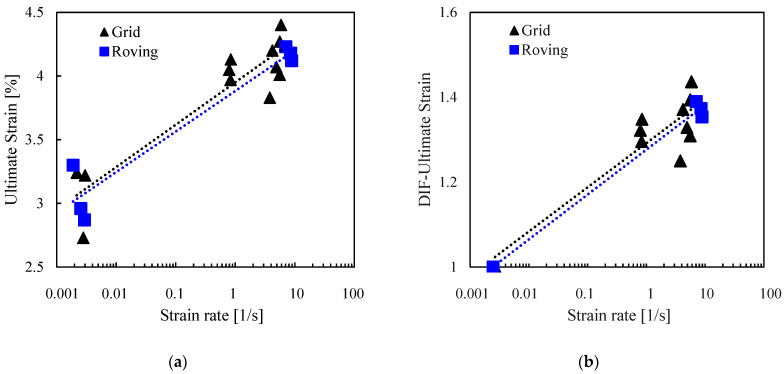
Effect of strain rate on (**a**) ultimate strain; (**b**) dynamic increase factor for ultimate strain.

Figure 9 illustrates the gradual increase in the ultimate strain with the increasing strain rate. The G10^−3^ and R10^−3^ specimens showed deformations ranging from 2.7% to 3.3% before rupture. In contrast, the dynamic specimens subjected to strain rates ranging from 1 to 8/s revealed a higher strain capacity, deforming by 3.8 to 4.4% before failure. This reflects a 30% increase in the strain capacity of the dynamic samples compared to the quasi-static ones. The results also showed that the specimen size did not significantly influence the ultimate strain of the roving and grid specimens under dynamic loading.

Studies on basalt fibre materials at higher strain rates have reported similar and differing failure strains and trends. Yao et al. [15] found that loading unidirectional basalt textiles at strain rates of 25–100/s increased the ultimate strain from 2.4 to 3.2%. In contrast, basalt filament tows tested between 600 and 3000/s decreased with the increasing strain rate and had an ultimate failure strain of less than 3% [16]. Regarding the effect of the specimen size on the ultimate strain, Ou et al. [10] showed that basalt FRP composites experienced 75% more ultimate strain than smaller basalt yarn specimens when tested in the range of 40–160/s.

#### 3.2.3. Toughness

**Figure 10:**
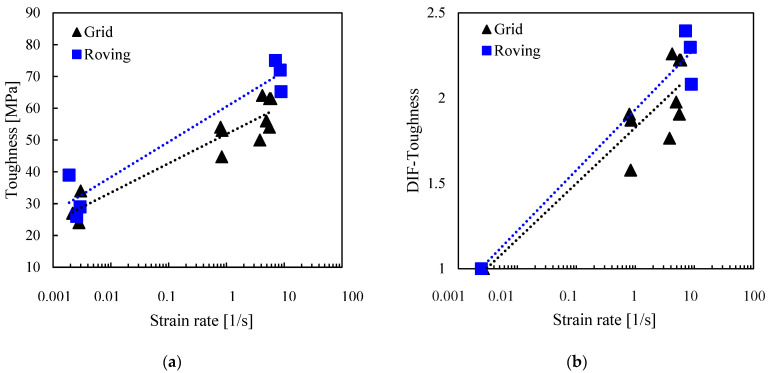
Effect of strain rate on (**a**) toughness; (**b**) dynamic increase factor for toughness.

Toughness was calculated using MATLAB’s ‘cumtrapz’ numerical integration function to find the area under the stress–strain curves, as shown in Figure 5. Figure 10 shows an overall increase in the energy absorbed by the material as the strain rate increased from 10^−3^ to 8/s. The average toughness of the grid at 5/s and roving at 8/s was more than twice that at 10^−3^. The graph also shows that the roving specimens absorbed more energy than the grid specimens. This observed trend is in agreement with the results reported in [44]. However, a contradictory study by [10] found that basalt grid specimens were 3.75 times tougher than the single yarn specimens at 40–160/s.

#### 3.2.4. Elastic Modulus

**Figure 11:**
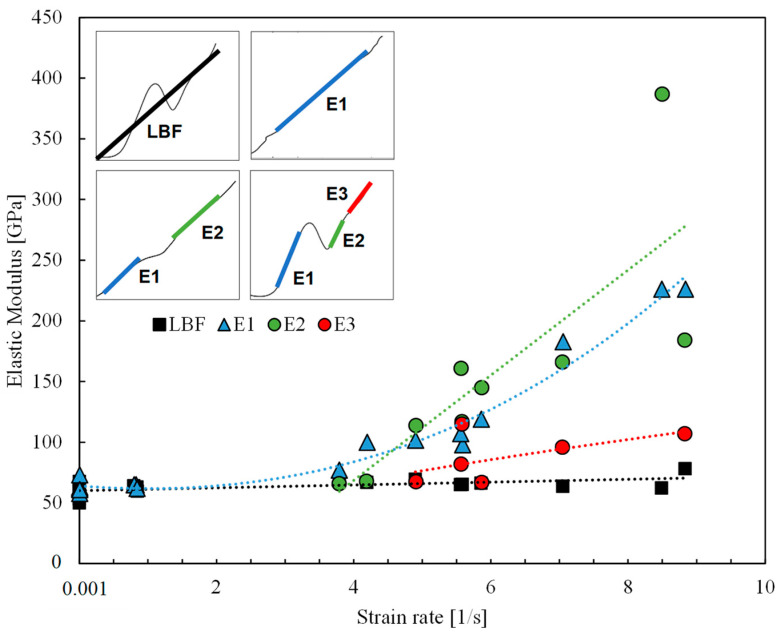
Effect of strain rate on elastic modulus.

Figure 11 shows the effect of the strain rate on the elastic modulus. The elastic modulus or stiffness was assessed in two ways. The first method (LBF) considered the slope of the best-fit line of each stress–strain curve in Figure 5a–e. The results of the LBF method demonstrated a moderate increase in stiffness (62.8 to 65 GPa) with an increasing strain rate from 10^−3^ to 8/s. The average stiffness was approximately 28% less than the manufacturer’s value. A similar methodology was employed by previous studies [10,15,45] to evaluate the elastic modulus of basalt tows, yarns, uni-directional textiles and BFRP composites at dynamic strain rates. Ou et al. [10] observed an increase in the elastic modulus of basalt yarn (62–87 GPa) at strain rates of 40–160/s and a relatively consistent stiffness of 62.5 GPa for BFRP composites over the same range. Uni-directional basalt textiles experienced increased stiffness (81.6 to 109.5 GPa) with increasing strain rates of 25–50/s but a reduced stiffness at 100/s. The stiffness of basalt tows at 600–3000/s also increased from 38 to 48 GPa. While the observed increase in stiffness in the present study was less pronounced, it corroborates the general trend that the stiffness of basalt fibre-based textiles is strain-rate sensitive.

The second approach evaluated the elastic moduli using the method of tangents [46]. This method involved identifying the linear regions of the stress–strain curves in Figure 5a–e, drawing a tangent to each region and calculating their slopes. The E1, E2, and E3 values in Figure 11 and Table 2 show a general trend of increasing stiffness with increasing strain rates in each region. The G4 specimens with two linear regions showed reduced stiffness in the second region compared with the first region (E1 > E2). In contrast, the G5 specimens with three regions showed the greatest stiffness in region 2 (E2 > E1 and E2 > E3). With respect to the roving specimens, the stiffness decreased with increasing linear regions, E1 > E2 > E3. E1 ranged from 62 to 119 GPa for the dynamic grid specimens and 182 to 226 GPa for the roving specimens. E2 for the grid ranged from 66 to 161 GPa, and for the single rovings, from 166 to 387 GPa. E3, the least stiff region, ranged from 65 to 115 GPa. The maximum stiffness values experienced by the grid and roving specimens were 145 and 387 GPa, respectively.

#### 3.2.5. Dynamic Increase Factor (DIF)

The dynamic increase factor was used to quantitatively describe the effect of the strain rate on the tensile strength, ultimate strain, and toughness of the basalt grid. The DIF is the ratio of the value under a dynamic strain rate to the average quasi-static value. Figure 8b, Figure 9b, and Figure 10b show the relationship between the DIFs and strain rates. Overall, DIFs were between 1.4 and 2.3. DIFs for the grid specimens at strain rates of 1 to 5/s ranged from 1.4 to 1.7 for tensile strength, from 1.3 to 1.4 for ultimate strain, and from 1.6 to 2.3 for toughness. For the R specimens tested at 8/s, the DIFs were in a similar range to that of the G specimens for the ultimate strain and toughness. However, the DIFs were higher for tensile strength (1.9 to 2.0).

The parameters of the DIF model, *DIF* = *A ln*($\dot{\varepsilon }$) + *B*, plotted in Figure 8b, Figure 9b and Figure 10b, are listed in Table 3. The reliabilities of the models were checked using Excel’s regression analysis tool, and the *p*-values associated with the ANOVA F statistic were also reported.

### 3.3. Strain Rate Effect on Tensile Failure

#### 3.3.1. Macroscopic Failure

**Figure 12:**
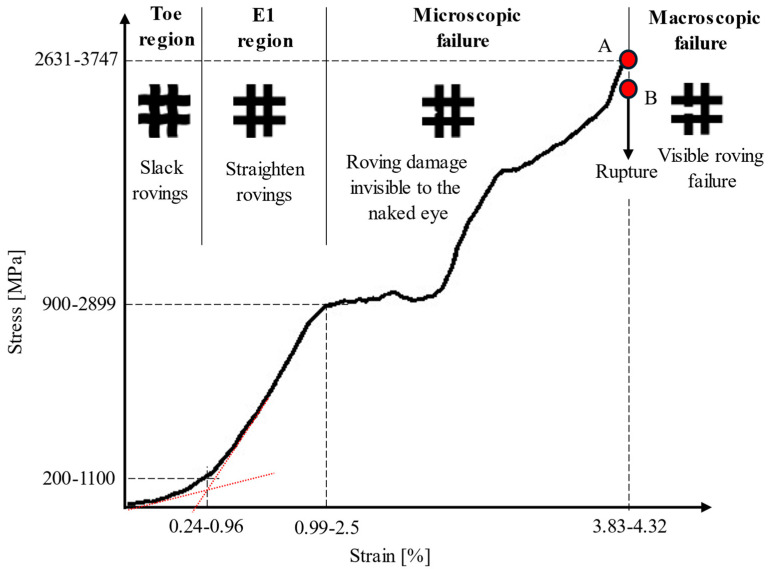
Schematic showing the basalt grid’s stress–strain response and different regions of failure progression under dynamic loading conditions.

**Figure 13:**
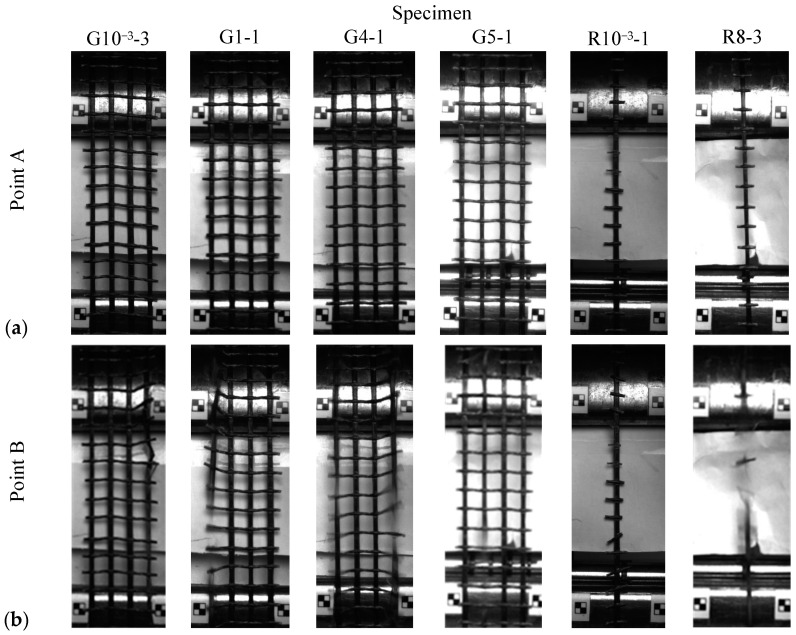
Image frame of representative specimens (**a**) Point A—ultimate tensile stress; (**b**) Point B—immediately after the point of ultimate tensile stress.

The G and R specimens experienced brittle and abrupt roving ruptures. Figure 12 illustrates the evolution of failure in the dynamic specimens in the range of 4 to 8/s in relation to their stress–strain responses. Initially, in the toe and E1 regions, the material underwent an adjustment process, during which the rovings were straightened. Beyond the E1 region, the microscopic deterioration of the individual fibres and polymer coatings began. This degradation progressed until the material reached its ultimate tensile stress, marked as point A. At this point, as shown in Figure 13a, no noticeable damage to the rovings was seen. However, shortly thereafter, at point B, the specimens exhibited macroscopic brittle failures as individual rovings ruptured (Figure 13b). For all samples, failure occurred within the gauge length region. Failures in the G specimens were initiated at arbitrary internal or external rovings. The random stress-concentrated areas in the grid were influenced by misalignment during testing, geometric irregularities, and material imperfections, as discussed in Section 3.1, Section 3.2.1 and Section 3.3.2, respectively.

#### 3.3.2. Microscopic Failure

After the tensile tests, SEM analysis was conducted on five samples (G10^−3^-1, G1-2, G4-2, G5-2, and R8-2) to examine the influence of the strain rate on the microscopic failure of the basalt grid. The micrographs showed that the basalt grid underwent fibre breakage, coating fracture and deformation, interfacial debonding between the fibres and coating, and delamination.

**Figure 14:**
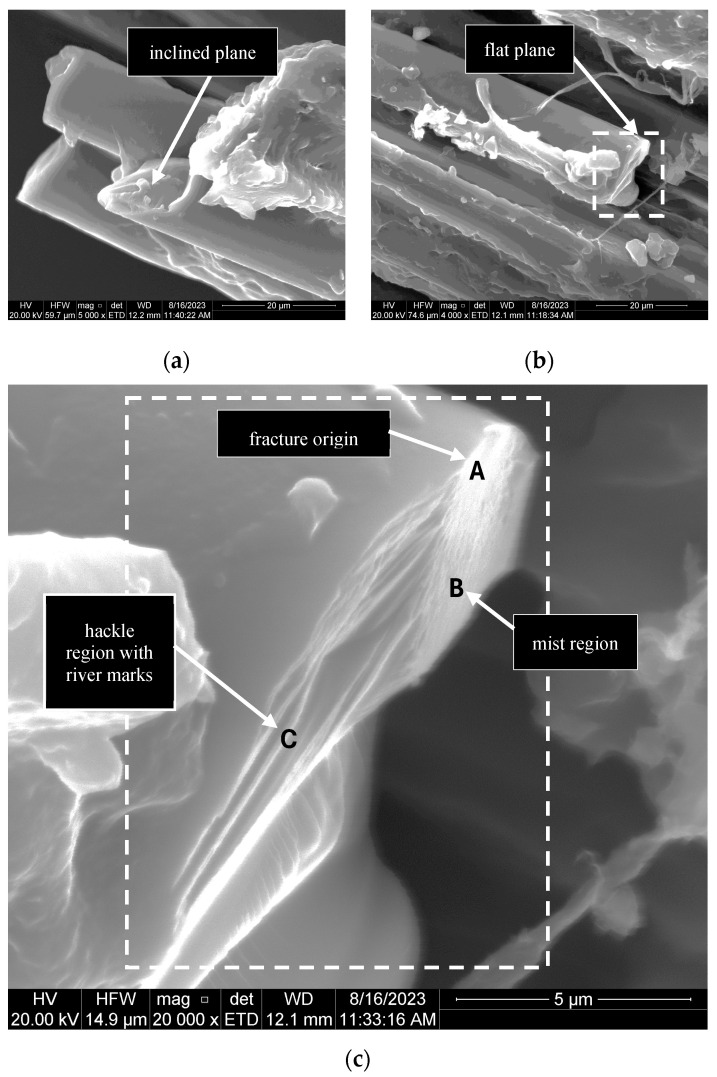
Morphology of the fibre fracture plane for G5-2: (**a**) inclined; (**b**) flat; and (**c**) magnification of the flat fracture plane showing features of a brittle fracture.

The basalt fibres experienced brittle failures at quasi-static and dynamic strain rates. The fracture planes of the fibres were predominantly flat (Figure 14b); inclined surfaces (Figure 14a) were observed only for G5-2 and R8-2. The flat fracture surface in Figure 14c exhibits the characteristic mirror, mist, and hackle features typically observed in brittle fibre fractures [48,49]. In this instance, the failure originated from the central area of the mirror region (A) and propagated throughout the mist region (B). The crack continued in the direction of the river markings in hackle region C until the fibre broke entirely.

**Figure 15:**
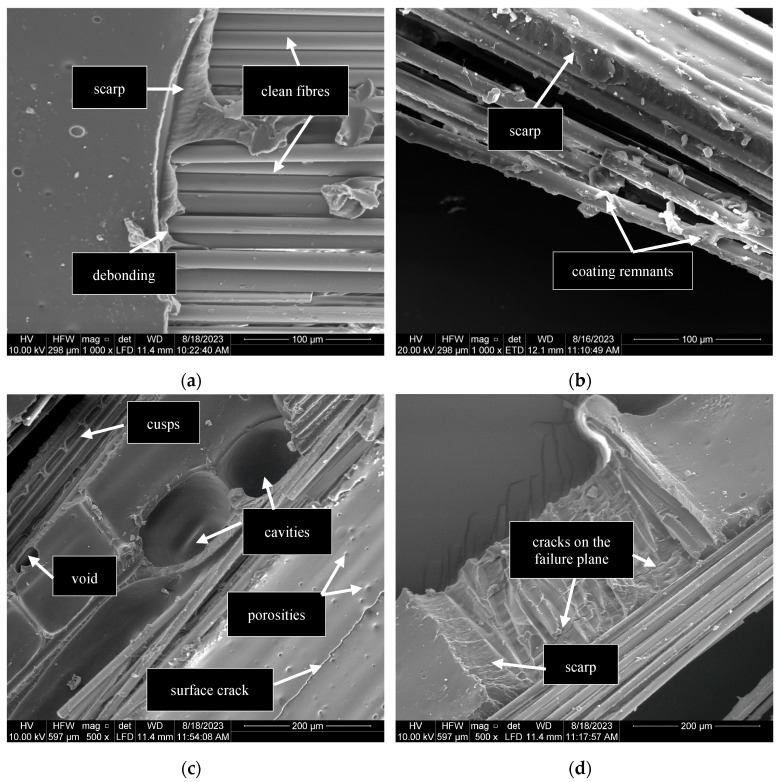
Brittle coating fracture at quasi-static and dynamic strain rates: (**a**) G10^−3^-1; (**b**) G5-2; and (**c**,**d**) R8-2.

**Figure 16:**
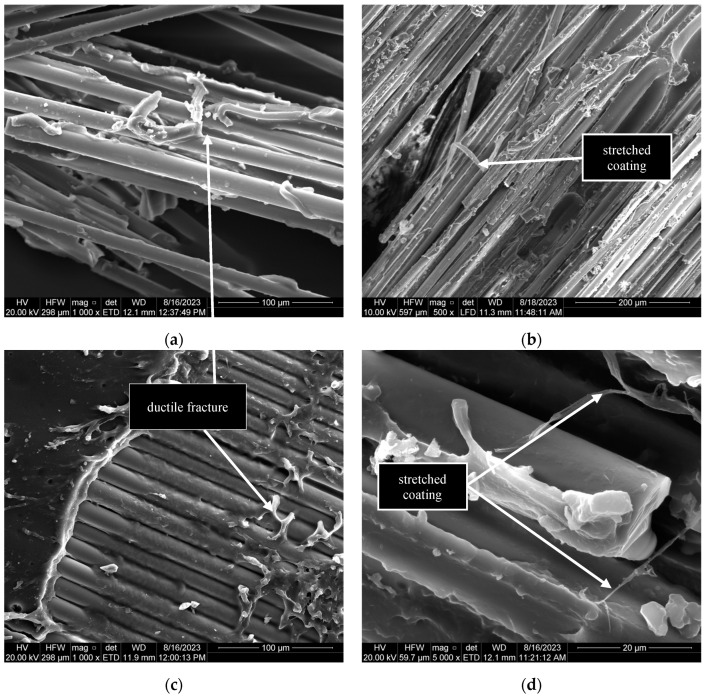
Ductile fracture of the coating at dynamic strain rates: (**a**) G4-2; (**b**) R8-2; and (**c**,**d**) G5-2.

Only brittle coating failure was observed in the quasi-static specimen G10^−3^-1 (Figure 15a); however, for dynamic specimens, a combination of brittle and ductile coating failures was observed (Figure 15 and Figure 16). Signs of brittle coating failures were scarps and cusps, which are commonly observed in brittle polymer matrices [50]. Figure 16 shows that the coating around the fibres was stretched and deformed at dynamic strain rates. A possible explanation for this observation might be the local softening of the coating in the area surrounding the fibre–coating interface [51]. It is inferred that the dynamic loading conditions caused a temperature increase in the immediate coating layer surrounding the fibres, resulting in the coating reaching its softening onset temperature. The heat generated would have been insufficient to soften the entire coating. Therefore, the external layer of the coating experienced brittle failure (Figure 15) as it would under quasi-static conditions. The non-linear stress–strain behaviour of the specimens at strain rates greater than 1/s was most likely due to the softening of the coating surrounding the fibres. Further research is required to validate this inference.

Failures in the coating were triggered and propagated by defects such as porosities, voids, cavities, and cracks, as shown in Figure 15. Microcracks were present on the external surface of the coating (Figure 15c) and along the failure plane (Figure 15d). However, it is uncertain whether these cracks occurred before or after failure. Figure 15c also shows that the coalescence of porosities played a role in the propagation of cracks. These defects may have resulted from the trapping of air during the impregnation process [52], the misalignment of fibres during impregnation, or inadequate bonding between the fibres and coating, as suggested by Gaylord [53]. Figure 15b provides evidence supporting the latter explanation, as it shows that the exterior coating layer has a thickness exceeding 30 μm (equivalent to the diameter of three fibres) and little to no coating between the fibres.

All five samples exhibited debonding, which is the separation or loss of adhesion between the fibres and the surrounding coating. Debonding weakens the stress transfer between the coating and the fibres, reducing the stiffness of the material. In addition, cracks can propagate effortlessly through the coating without the fibres, reducing toughness [54]. Evidence of this phenomenon can be observed for the stress–strain behaviour of the dynamic specimens, where the stiffness, E1 to E3 (Figure 11), progressively decreased with the increasing tensile load. Debonding could also explain why the toughness of G1-2 was 15% less than that of G1-1 and G1-3.

**Figure 17:**
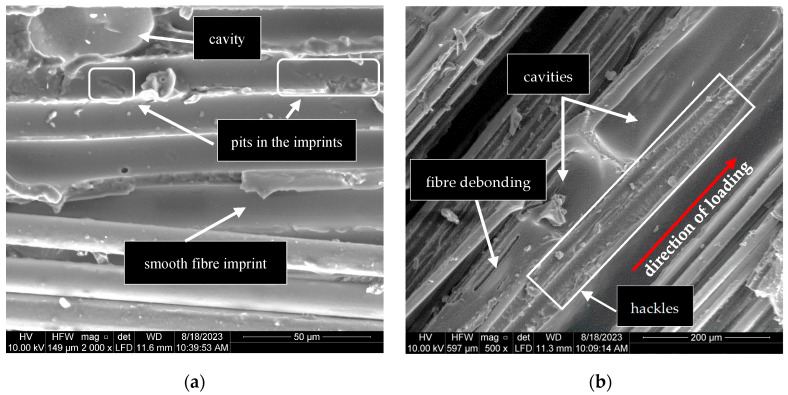
Delamination fracture surfaces: (**a**) G10^−3^-1; (**b**) R8-2.

The micrographs revealed variations in the interfacial bond strength between the fibres and coating. Generally, the surfaces of the debonded fibres appeared rough, with remnants of the coating still attached (Figure 15 and Figure 16), suggesting strong adhesion between the fibres and the coating. However, sample G10^−3^-1 in Figure 15a demonstrated weak interfacial bonding, as indicated by the absence of the coating on the fibres [55]. Additionally, the smooth and pitted fibre imprints observed within the coating (Figure 17) further suggested inconsistencies in the interfacial bond strength.

The fracture surfaces shown in Figure 17 highlight two prominent features of delamination, fibre imprints and hackles, as previously noted by [56]. The hackles oriented perpendicular to the loading direction in Figure 17b are suggestive of a mode II fracture within the roving specimen, as described in [57]. The large cavities on the fracture surfaces are weak points in the coating, which promote delamination. Delamination was observed in specimens G10^−3^-1 and R8-2, which exhibited unusual stress–strain behaviours. These observations indicate that cavities and delamination in the coating significantly influence the stress–strain response of the grid under quasi-static and dynamic strain rates.

## 4. Conclusions

The tensile characterisation of the basalt fibre grid was successfully conducted at intermediate (1 to 8/s) and quasi-static (10^−3^/s) strain rates using specialised grips developed for this purpose. The key conclusions drawn from this study are highlighted below.

- The type of grip and specimen used for the dynamic tensile testing of the material are essential for achieving valid results. Initial approaches, including clamp grips and SG1 specialised grip, failed due to slippage, non-uniform stress distribution, and premature failures. The modified SG2 grips, featuring vice-type clamps and reduced gauge length, eliminated slippage and enabled consistent strain rates and valid specimen failures.
- The basalt grid displayed a linear stress–strain relationship in the range of 10^−3^ to 1/s. Conversely, non-linear stress–strain relationships with several linear and transition regions were observed when the strain rate ranged from 4 to 8/s. Specimens with anomalous stress–strain behaviours were found to experience misalignment during testing.
- An increased strain rate enhanced the tensile strength, ultimate strain, toughness, and elastic modulus of the basalt grid. This renders the grid promising for retrofitting structures that may experience dynamic loadings such as vibrations or impacts.
- The correlation between the strain rate and tensile strength, ultimate strain, and toughness was logarithmic. Overall, the dynamic increase factors ranged between 1.4 and 2.3. The DIF for the tensile strength, ultimate strain and toughness in the range of 10^−3^ and 8/s can be predicted with a 10% confidence level using the following model: *DIF* = *A ln*($\dot{\varepsilon }$) + *B*.
- The grid specimens reached lower strain rate values than single rovings at the same loading rate of 1100 mm/s; however, in the quasi-static range (1 mm/s), the specimen size did not affect the strain rate experienced by the samples. The single-roving specimens exhibited higher tensile strength, toughness, and elastic modulus than the larger specimens. However, the ultimate strains were comparable for both specimen sizes. The different grid and roving behaviours can be attributed to the non-uniform stress distribution in the grid caused by the geometric roving misalignment and grid effect.
- At the macroscopic level, the basalt grid generally experienced brittle failure. Microscopic investigation revealed that the failure mechanisms included fibre and coating fractures, debonding, and delamination. The SEM analyses also showed that the strain rate influenced the angle of the basalt fibres’ fracture plane and the ductility of the polymer coating.
- Defects such as cavities, voids, and microcracks were found on the fracture surfaces of the samples, especially those that displayed anomalous stress–strain behaviour and reduced toughness and stiffness. Another critical issue was the insufficient coating around the fibres, which compromised the interfacial bond strength of the fibres and coating.

Further research is required to address the issue of grid misalignment during testing. It would also be useful to investigate the effect of the strain rate on the individual components of the grid, including the fibre, coating, and interface. Finally, improved coating techniques should be explored to reduce defects and mitigate fibre debonding. Improving the polymer coating can significantly improve the durability and load-bearing capacity of TRMs, particularly under dynamic loading conditions.

## Figures and Tables

**Table 1:** Summary of specimen ID, specimen geometry, machine displacement rate, and measured strain rate.

Specimen ID	Specimen Geometry			Machine Displacement Rate [mm/s]	Measured Strain Rate [1/s]
	# of Rovings	Width [mm]	Total Length [mm]		
Grid					
G10^−3^-1	4	26.8	490		2.2 × 10^−3^
G10^−3^-2	4	26.8	490	1	2.8 × 10^−3^
G10^−3^-3	4	26.8	490		3.0 × 10^−3^
G1-1	4	26.8	490		0.79
G1-2	4	26.8	490	200	0.83
G1-3	4	26.8	490		0.84
G4-1	4	26.8	490	800	3.79
G4-2	4	26.8	490		4.19
G5-1	4	26.8	490	1100	4.90
G5-2	4	26.8	490		5.58
G5-3	4	26.8	490		5.56
G5-4	4	26.8	490		5.86
Roving					
R10^−3^-1	1	6.7	490	1	1.9 × 10^−3^
R10^−3^-2	1	6.7	490		2.5 × 10^−3^
R10^−3^-3	1	6.7	490		2.9 × 10^−3^
R8-1	1	6.7	490	1100	7.05
R8-2	1	6.7	490		8.49
R8-3	1	6.7	490		8.83

**Table 2:** Elastic modulus of grid and roving specimens.

Specimen ID	Elastic Modulus [GPa]			
	LBF	E1	E2	E3
Grid				
G10^−3^-1	50.0	62.0		
G10^−3^-2	60.9	62.0		
G10^−3^-3	60.7	63.0		
G1-1	64.1	65.0		
G1-2	63.1	65.0		
G1-3	59.7	62.0		
G4-1	67.7	77.0	66.0	
G4-2	66.9	100.0	68.0	
G5-1	69.6	101.8	113.9	67.5
G5-2	65.4	98.0	117.0	115.0
G5-3	65.2	107.0	161.0	82.0
G5-4	66.4	119.0	145.0	67.0
Roving				
R10^−3^-1	67.8	73.2		
R10^−3^-2	53.4	57.9		
R10^−3^-3	61.6	61.1		
R8-1	63.8	182.8	166.2	96.0
R8-2	62.4	226.1	387.0	
R8-3	78.3	226.1	184.2	107.0

**Table 3:** DIF model parameters for strength, ultimate strain, and toughness at 5% CL, where *DIF* = *A ln*($\dot{\varepsilon }$) + *B*.

Tensile Property	Specimen Type	Parameters		*p*-Value
		A	B	
Tensile strength [MPa]	grid	0.0806	1.4564	0.0036
	roving	0.113	1.6796	0.0265
Ultimate strain [%]	grid	0.0452	1.2919	0.0710 *
	roving	0.0459	1.2763	0.0343
Toughness [MPa]	grid	0.1424	1.8276	0.0178
	roving	0.1545	1.933	0.0704 *
* Model is statistically insignificant				

The tensile properties of the grid and roving specimens were all statistically significant at the 5% confidence level (CL), except for the grid’s ultimate strain and roving specimens’ toughness models. However, these models were statistically significant at the 10% CL. Similar models were proposed by [44,47] for unidirectional and basalt FRPs; however, no models for TRM grids are available in the literature.

## Data Availability

The original contributions presented in the study are included in the article, further inquiries can be directed to the corresponding author.

## References

[1] Ritchie P.A. (1988). External Reinforcement of Concrete Beams Using Fiber Reinforced Plastic. Master’s Thesis.

[2] Triantafillou T., Plevris N. (1992). Strengthening of RC beams with epoxy-bonded fibre-composite materials. Mater. Struct..

[3] Triantafillou T.C., Papanicolaou C.G. (2005). Textile Reinforced Mortars (TRM) versus Fiber Reinforced Polymers (FRP) as Strengthening Materials of Concrete Structures. Spec. Publ..

[4] Mazzuca P., Pisani B., Firmo J.P., Ombres L. (2024). Tensile and bond properties at elevated temperatures of a PBO-FRCM composite system for strengthening concrete elements: Experimental and analytical investigations. Constr. Build. Mater..

[5] Buchan P.A., Chen J.F. (2007). Blast resistance of FRP composites and polymer strengthened concrete and masonry structures—A state-of-the-art review. Compos. Part B Eng..

[6] George J.M., Kimiaei M., Elchalakani M., Fawzia S. (2022). Underwater strengthening and repairing of tubular offshore structural members using Carbon Fibre Reinforced Polymers with different consolidation methods. Thin-Walled Struct..

[7] Lin H., Han C., Yang L., Karampour H., Luan H., Han P., Xu H., Zhang S. (2022). Dynamic Performance and Crashworthiness Assessment of Honeycomb Reinforced Tubular Pipe in the Jacket Platform under Ship Collision. J. Mar. Sci. Eng..

[8] Zhou Y., Wang X., Hu B., Sui L., Yuan F. (2023). Seismic Retrofit of Nonuniformly Corroded Coastal Bridge Piers with FRP and Engineered Cementitious Composite Overlays. J. Compos. Constr..

[9] Dhakal A., Parvin A. (2021). Fiber Reinforced Polymer as Wood Roof-to-Wall Connections to Withstand Hurricane Wind Loads. Civ. Eng..

[10] Ou Y., Zhu D., Li H. (2016). Strain rate and temperature effects on the dynamic tensile behaviors of basalt fiber bundles and reinforced polymer composite. J. Mater. Civ. Eng..

[11] Bai Y.-L., Yan Z.-W., Ozbakkaloglu T., Han Q., Dai J.-G., Zhu D.-J. (2020). Quasi-static and dynamic tensile properties of large-rupture-strain (LRS) polyethylene terephthalate fiber bundle. Constr. Build. Mater..

[12] Zhu D., Peled A., Mobasher B. (2011). Dynamic tensile testing of fabric–cement composites. Constr. Build. Mater..

[13] Ou Y., Zhu D., Huang M., Li H. (2017). The effects of gage length and strain rate on tensile behavior of Kevlar^®^ 29 single filament and yarn. J. Compos. Mater..

[14] Ou Y., Zhu D., Zhang H., Huang L., Yao Y., Li G., Mobasher B. (2016). Mechanical Characterization of the Tensile Properties of Glass Fiber and Its Reinforced Polymer (GFRP) Composite under Varying Strain Rates and Temperatures. Polymers.

[15] Yao Y., Zhu D., Zhang H., Li G., Mobasher B. (2016). Tensile behaviors of basalt, carbon, glass, and aramid fabrics under various strain rates. J. Mater. Civ. Eng..

[16] Zhu L., Sun B., Gu B. (2012). Frequency features of basalt filament tows under quasi-static and high strain rate tension. J. Compos. Mater..

[17] Milling A., Amato G., Taylor S., Robinson D. Experimental and numerical analysis of the tensile behaviour of basalt textile at various strain rates. Proceedings of the 11th International Conference on Fiber-Reinforced Polymer (FRP) Composites in Civil Engineering (CICE 2023).

[18] Ahmed A., Rahman M.Z., Ou Y., Liu S., Mobasher B., Guo S., Zhu D. (2021). A review on the tensile behavior of fiber-reinforced polymer composites under varying strain rates and temperatures. Constr. Build. Mater..

[19] Vemuganti S., Soliman E., Reda Taha M. (2020). 3D-printed pseudo ductile fiber-reinforced polymer (FRP) composite using discrete fiber orientations. Fibers.

[20] Zhang S. (2018). Intermediate Strain Rate Behaviour of Pultruded Glass Fibre Reinforced Polymer (GFRP). Ph.D. Thesis.

[21] Crocco M.C., Scuro C., Filosa R., Codispoti R., Ferraro M., Solano A., Agostino R.G., Barberi R.C., Olivito R.S., Formoso V. (2023). Experimental Study on the Mechanical Properties of Basalt FRCM Made of Various Matrices: Validation by X-Ray Microtomography. J. Mater. Civ. Eng..

[22] Bhat T., Chevali V., Liu X., Feih S., Mouritz A. (2015). Fire structural resistance of basalt fibre composite. Compos. Part A Appl. Sci. Manuf..

[23] Militký J., Mishra R., Jamshaid H., Bunsell A.R. (2018). 20—Basalt fibers. Handbook of Properties of Textile and Technical Fibres.

[24] Fiore V., Scalici T., Di Bella G., Valenza A. (2015). A review on basalt fibre and its composites. Compos. Part B Eng..

[25] Wang W., Zhang Y., Mo Z., Chouw N., Jayaraman K., Xu Z.-D. (2023). A critical review on the properties of natural fibre reinforced concrete composites subjected to impact loading. J. Build. Eng..

[26] Liu H., Yu Y., Liu Y., Zhang M., Li L., Ma L., Sun Y., Wang W. (2022). A Review on Basalt Fiber Composites and Their Applications in Clean Energy Sector and Power Grids. Polymers.

[27] Oddo M.C., Minafò G., La Mendola L. (2023). Experimental investigation on tensile and shear bond behaviour of Basalt-FRCM composites for strengthening calcarenite masonry elements. Procedia Struct. Integr..

[28] Oddo M.C., Cavaleri L., Papanicolaou C., La Mendola L. (2024). Experimental Characterization of Fabric-Reinforced Cementitious Matrix (FRCM) Systems Applied on Calcarenite Stone: Adoption of Non-Standard Setup for Double-Shear Bond Tests. J. Compos. Sci..

[29] D’Anna J., Amato G., Chen J.F., Minafò G., La Mendola L. (2021). Experimental application of digital image correlation for the tensile characterization of basalt FRCM composites. Constr. Build. Mater..

[30] Lydon D., Lydon M., Del Rincon J.M., Taylor S.E., Robinson D., O’Brien E., Catbas F.N. (2018). Development and field testing of a time-synchronized system for multi-point displacement calculation using low-cost wireless vision-based sensors. IEEE Sens. J..

[31] Luyckx T., Verstraete M., De Roo K., De Waele W., Bellemans J., Victor J. (2014). Digital image correlation as a tool for three-dimensional strain analysis in human tendon tissue. J. Exp. Orthop..

[32] Stepanović J., Ćirković N., Sadiković A., Stepanović J. (2024). Analysis of the deformation characteristics of woven textile materials in plain weave. Adv. Technol..

[33] Burkinshaw S.M. (2016). Physico-Chemical Aspects of Textile Coloration.

[34] Hughes M., Carpenter J., Hill C. (2007). Deformation and fracture behaviour of flax fibre reinforced thermosetting polymer matrix composites. J. Mater. Sci..

[35] Zhou G., Sun Q., Meng Z., Li D., Peng Y., Zeng D., Su X. (2021). Experimental investigation on the effects of fabric architectures on mechanical and damage behaviors of carbon/epoxy woven composites. Compos. Struct..

[36] Bogdanovich A.E., Karahan M., Lomov S.V., Verpoest I. (2013). Quasi-static tensile behavior and damage of carbon/epoxy composite reinforced with 3D non-crimp orthogonal woven fabric. Mech. Mater..

[37] Tamrakar S., Ganesh R., Sockalingam S., Haque B.Z., Gillespie J.W. (2020). Strain rate-dependent large deformation inelastic behavior of an epoxy resin. J. Compos. Mater..

[38] Yang Y., Yu J., Xu H., Sun B. (2017). Porous Lightweight Composites Reinforced with Fibrous Structures.

[39] Broughton R.M., Mogahzy Y.E., Hall D. (1992). Mechanism of yarn failure. Text. Res. J..

[40] Wang Y., Miao Y., Huang L., Swenson D., Yen C.-F., Yu J., Zheng J.Q. (2016). Effect of the inter-fiber friction on fiber damage propagation and ballistic limit of 2-D woven fabrics under a fully confined boundary condition. Int. J. Impact Eng..

[41] Briscoe B., Motamedi F. (1992). The ballistic impact characteristics of aramid fabrics: The influence of interface friction. Wear.

[42] Gilat A., Goldberg R.K., Roberts G.D. (2007). Strain rate sensitivity of epoxy resin in tensile and shear loading. J. Aerosp. Eng..

[43] Fitoussi J., Bocquet M., Meraghni F. (2013). Effect of the matrix behavior on the damage of ethylene–propylene glass fiber reinforced composite subjected to high strain rate tension. Compos. Part B Eng..

[44] Xu X., Rawat P., Shi Y., Zhu D. (2019). Tensile mechanical properties of basalt fiber reinforced polymer tendons at low to intermediate strain rates. Compos. Part B Eng..

[45] Zhu D., Ou Y. (2016). Strain rate and temperature effects on mechanical behavior of BFRP single yarns. J. Build. Mater.

[46] (2017). Standard Test Method for Young’s Modulus, Tangent Modulus, and Chord Modulus.

[47] Chen W., Hao H., Jong M., Cui J., Shi Y., Chen L., Pham T.M. (2017). Quasi-static and dynamic tensile properties of basalt fibre reinforced polymer. Compos. Part B Eng..

[48] Kessler E., Gadow R., Straub J. (2016). Basalt, glass and carbon fibers and their fiber reinforced polymer composites under thermal and mechanical load. AIMS Mater. Sci..

[49] Hearle J., Elices M., Llorca J. (2002). Forms of fibre fracture. Fiber fracture.

[50] Greenhalgh E. (2009). Failure Analysis and Fractography of Polymer Composites.

[51] Taniguchi N., Nishiwaki T., Hirayama N., Nishida H., Kawada H. (2009). Dynamic tensile properties of carbon fiber composite based on thermoplastic epoxy resin loaded in matrix-dominant directions. Compos. Sci. Technol..

[52] Judd N.C.W., Wright W.W. (1978). Voids and their effects on the mechanical properties of composites—An Appraisal. SAMPE J..

[53] Gaylord M. (1974). Reinforced Plastics: Theory and Practice.

[54] Zhang Z., Hartwig G. (2002). Relation of damping and fatigue damage of unidirectional fibre composites. Int. J. Fatigue.

[55] Deng S., Ye L. (2000). Influence of fibre-matrix adhesion on mechanical properties of graphite/epoxy composites: III. Impact and dynamic mechanical properties. J. Reinf. Plast. Compos..

[56] Alderliesten R. (2013). Critical review on the assessment of fatigue and fracture in composite materials and structures. Eng. Fail. Anal..

[57] Marat-Mendes R., de Freitas M. Characterization of Delamination Fracture Surfaces Under Mixed Mode Loading. Proceedings of the Iberian Conference on Fracture and Structural Integrity.

